# Duration of Chemotherapy for Small Cell Lung Cancer: A Meta-Analysis

**DOI:** 10.1371/journal.pone.0073805

**Published:** 2013-08-30

**Authors:** Hang Zhou, Chao Zeng, Yang Wei, Jin Zhou, Wenxiu Yao

**Affiliations:** 1 Department of Chemotherapy, Sichuan Cancer Hospital, Chengdu, China; 2 Department of Gastroenterology, the Third People's Hospital of Chengdu, Chengdu, China; Queen Elizabeth Hospital, Hong Kong

## Abstract

**Background:**

Maintenance chemotherapy is widely provided to patients with small cell lung cancer (SCLC). However, the benefits of maintenance chemotherapy compared with observation are a subject of debate.

**Methodology and Principal Findings:**

To identify relevant literature, we systematically searched the Medline, Embase, and Cochrane Central Register of Controlled Trials databases. Eligible trials included patients with SCLC who either received maintenance chemotherapy (administered according to a continuous or switch strategy) or underwent observation. The primary outcome was 1-year mortality, and secondary outcomes were 2-year mortality, overall survival (OS), and progression-free survival (PFS). Of the 665 studies found in our search, we identified 14 relevant trials, which together reported data on 1806 patients with SCLC. When compared with observation, maintenance chemotherapy had no effect on 1-year mortality (odds ratio [OR]: 0.88; 95% confidence interval [CI]: 0.66–1.19; P = 0.414), 2-year mortality (OR: 0.82; 95% CI: 0.57–1.19; P = 0.302), OS (hazard ratio [HR]: 0.87; 95% CI: 0.71–1.06; P = 0.172), or PFS (HR: 0.87; 95% CI: 0.62–1.22; P = 0.432). However, subgroup analyses indicated that maintenance chemotherapy was associated with significantly longer PFS than observation in patients with extensive SCLC (HR, 0.72; 95% CI: 0.58–0.89; P = 0.003). Additionally, patients who were managed using the continuous strategy of maintenance chemotherapy appeared to be at a disadvantage in terms of PFS compared with patients who only underwent observation (HR, 1.27; 95% CI: 1.04–1.54; P = 0.018).

**Conclusions/Significance:**

Maintenance chemotherapy failed to improve survival outcomes in patients with SCLC. However, a significant advantage in terms of PFS was observed for maintenance chemotherapy in patients with extensive disease. Additionally, our results suggest that the continuous strategy is inferior to observation; its clinical value needs to be investigated in additional trials.

## Introduction

Small cell lung cancer (SCLC), which accounts for approximately 20% of all lung cancer cases, has a high growth fraction and is often widely metastatic [1]–[2]. The standard of first-line chemotherapy for SCLC currently depends on the extent of disease at diagnosis [3]. High response rates and substantially prolonged survival have been achieved by combination chemotherapy with or without thoracic radiation therapy [4]–[5]. However, no significant improvements in survival have been observed for SCLC patients who receive maintenance chemotherapy [6]–[8]. We evaluated the effects of chemotherapy on survival outcomes for patients with SCLC, including maintenance chemotherapy with the same regimens used during induction treatment (the continuous strategy) as well as chemotherapy that involved other agents (the switch strategy).

Historically, standard chemotherapy has provided modest improvements to overall survival (OS) and progression-free survival (PFS) for patients with SCLC. Patients treated with chemotherapy have also reported better quality of life, as measured by their scores on quality of life functional scales [9]–[13]. However, it remains unclear whether maintenance chemotherapy is more effective than observation for patients with SCLC. A previous meta-analysis [14] showed that maintenance and consolidation therapy both failed to improve survival outcomes for patients with SCLC. Although a slight survival advantage was detected for maintenance chemotherapy, the difference was not statistically significant. To investigate maintenance therapy specifically and in greater detail, we carried out a systematic review and meta-analysis of pooled data from randomized controlled trials that evaluated the effects of maintenance chemotherapy on the survival of patients with SCLC.

## Methods

### Data sources, search strategy, and selection criteria

This review was conducted and reported according to the Preferred Reporting Items for Systematic Reviews and Meta-Analysis (PRISMA) Statement issued in 2009 [15] (Table S1). All English-language randomized controlled trials of maintenance chemotherapy were eligible for inclusion in our meta-analysis, as long as they examined the efficacy of maintenance chemotherapy on 1-year mortality, 2-year mortality, OS, or PFS. Trials were eligible for inclusion regardless of their publication status (published, unpublished, in press, or in progress). Relevant trials were identified according to the following procedures:

1. Electronic searches: We searched the Medline, Embase, and Cochrane Central Register of Controlled Trials electronic databases for articles published between 1950 and November 2012, using “SCLC” or “small cell lung cancer” or “carcinoma and small lung cancer” AND (“maintenance” OR “consolidation” AND “antineoplastic agents”) as the search terms. The reference lists from all reports on non-randomized controlled trials were also searched manually to identify additional eligible studies.
2. Other sources: We contacted authors to obtain any possible additional published or unpublished data. We additionally searched the websites of http://www.who.int/trialsearch and http://www.ClinicalTrials.gov for information on registered randomized controlled trials. The medical subject headings, methods, patient population, interventions, and outcomes variables of these studies were used to identify relevant trials.

The literature search, data extraction, and quality assessment were independently undertaken by 3 authors (HZ, CZ, and YW), following a standardized approach. Any inconsistencies between these authors were settled by the primary author (WY). Studies were eligible for inclusion if they met the following criteria: (1) the study included patients with SCLC; (2) the study was a randomized controlled trial; (3) the trial evaluated the efficacy of maintenance chemotherapy compared to observation; and (4) the trial reported at least 1 outcome as 1-year mortality, 2-year mortality, OS, or PFS.

### Data collection and quality assessment

All data from eligible trials were abstracted, independently and in duplicate, by 2 investigators (HZ and YW) according to a standard protocol. Subsequently, the data were reviewed by a third investigator (JZ). Any discrepancies were resolved through group discussion. The collected data included several baseline characteristics: the first author or study group name, the year of publication, the number of patients enrolled, the mean age, the proportion of patients who were male, the interventions, and the duration of follow-up. One-year mortality, 2-year mortality, OS, and PFS were investigated as outcomes. Study quality was assessed using the Jadad Score [16], which is based on 5 subscales: randomization (1 or 0), concealment of the treatment allocation (1 or 0), blinding (1 or 0), completeness of follow-up (1 or 0), and the use of an intention-to-treat analysis (1 or 0). These subscales are combined to produce a scoring system ranging from 0 to 5. In our study, we considered trials awarded scores of 3 or greater as high-quality studies.

### Statistical analysis

The efficacy of maintenance chemotherapy was estimated by hazard ratios (HRs) and odds ratios (ORs), with corresponding 95% confidence intervals (CIs). For time-to-event data (OS and PFS), log hazard ratios and their variances were estimated using the methods of Parmar [17] when CIs of HRs were available. The summary HRs and their 95% CIs were estimated according to a general variance-based method. For 1-year mortality and 2-year mortality, the estimates of treatment effects were obtained from the event numbers, which were extracted from each trial and combined using the Mantel–Haenszel method. Both fixed-effects and random-effects models were used to assess pooled HRs and ORs for maintenance chemotherapy compared with observation. Although both models yielded similar findings, the results from the random-effects model presented here assume that the true underlying effect varies among included trials [18]–[19].

The heterogeneity of treatment effects between studies was investigated visually using scatter plot analysis as well as statistically using the heterogeneity I^2^ statistic [20]–[21]. Subgroup analyses were conducted on the basis of publication years, number of patients, stage of disease, and the regimen of maintenance chemotherapy (continuous or switch strategy) to minimize the consequences of heterogeneity among the included trials and, additionally, to evaluate the efficacy of maintenance therapy in specific subpopulations. We also performed sensitivity analysis by removing each individual trial from the meta-analysis and reapplying our statistical methods, to the stability of our results.

We employed several methods to check for potential publication bias. We conducted visual inspections of funnel plots for 1-year mortality, 2-year mortality, OS, and PFS. Additionally, the Egger test [22] and Begg test [23] were used to check for potential publication bias. All reported P-values are 2-sided and values of P<0.05 were regarded as significant for all included studies. Statistical analyses were performed using STATA software (version 10.0).

## Results

### Trial characteristic

**Figure 1:**
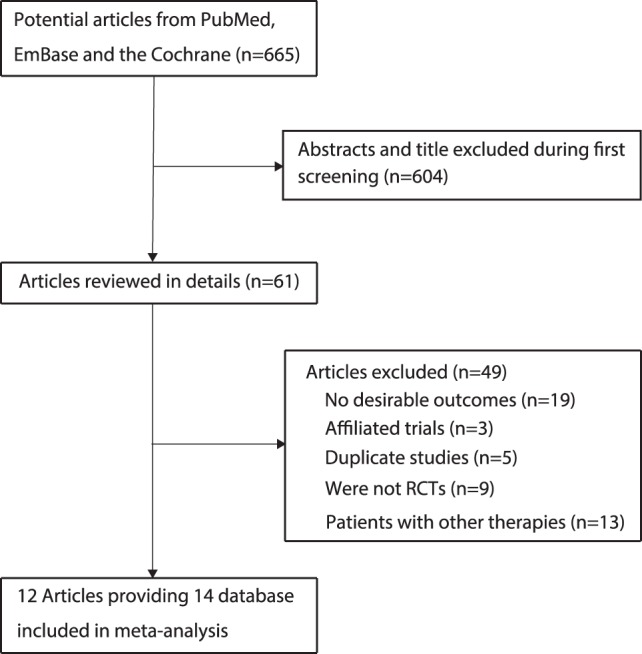
Flow diagram of the literature search and trials selection process.

We identified 665 articles in our initial electronic search, of which 604 were excluded during an initial review of titles and abstracts. We retrieved the full text for the remaining 61 articles, and 14 subpopulations [6]–[8], [10]–[13], [24]–[28] met the inclusion criteria (Figure 1). Table 1 summarizes the characteristics of these trials and key baseline information on the 1806 included patients with SCLC. The trials included in this study compared maintenance chemotherapy to observation in terms of 1-year mortality, 2-year mortality, OS, or PFS. The number of patients included in each trial ranged from 32 to 434. Nine of the included trials [6]–[8], [10], [24], [25], [28] evaluated the continuous strategy as maintenance chemotherapy, and the remaining 5 trials [11]–[13], [26]–[27] assessed the switch strategy as maintenance chemotherapy. The numbers of trials including 1-year mortality [6]–[8], [10]–[13], [24]–[28], 2-year mortality [6]–[8], [10]–[13], [24]–[28], OS [6]–[8], [10]–[13], [24]–[27], and PFS [8], [10]–[12], [25]–[27] were 14, 14, 13, and 8, respectively. Jadad scores [16] were used to assess the quality of the included trials. Overall, 7 trials [6], [8], [10]–[12], [25] had a Jadad score of 3, 4 trials [13], [24], [27] had a score of 2, and the remaining 3 trials [7], [26], [28] had a score of 1.

**Table 1:** Design and characteristic of trials included in our meta-analysis.

Source	No. of patients	Stage of disease	Induction chemotherapy	Maintenance chemotherapy	Jadad score
Byrne MJ (1989) [6]	66	Limited disease	Cisplatinum and VP16213 q for 3 weeks followed by cyclophosphamide, vincristine and methotrexate (CVM)q. 4 weeks.	Cyclophosphamide, vincristine and methotrexate for additional 6 cycles; no treatment	3
Bleehen NM (1989) [7]	265	Limited disease and extensive disease	Etoposide, cyclophosphamide, methotrexate and vincristine for 6 cycles	Etoposide, cyclophosphamide, methotrexate and vincristine for 6 cycles; no treatment	1
DS Ettinger (1990) [8]	36	Extensive disease	Cyclophosphamide, doxorubicin, vincristine-altretamine for 6–8 cycles	Cyclophosphamide, doxorubicin, vincristine -altretamine up to 28 cycles; no treatment	3
DS Ettinger (1990) [8]	50	Extensive disease	Cyclophosphamide, doxorubicin, vincristine-altretamine (hexamethylmelamine), etoposide, and methotrexate for 6–8 cycles	Cyclophosphamide, doxorubicin, vincristine -altretamine (hexamethylmelamine), etoposide, and methotrexate up to cycles; no treatment	3
G Giaccone (1993) [10]	434	Limited disease and extensive disease	Cyclophosphamide, doxorubicin, and etoposide for 5 cycles	cyclophosphamide, doxorubicin, and etoposide for additional 7 cycles; no treatment	3
JP Sculier (1996) [11]	91	Limited disease and extensive disease	Ifosfamide, etoposide, and an anthracycline (doxorubicin or epirubicin) for 6 cycles	Etoposide and vindesine for additional 12 cycles; no treatment	3
JH Schiller (2001) [12]	223	Extensive disease	Cisplatin and etoposide for 4 cycles	Topotecan for additional 4 cycles; no treatment	3
DH Johnson (1993) [13]	151	Limited disease	Cyclophosphamide, doxorubicin, and vincristine for 6 cycles, plus radiotherapy	cisplatin plus etoposide for 2 cycles; no treatment	2
Midlands Small Cell Lung Cancer Group (1986) [24]	61	Extensive disease	Vincristine, doxorubicin, and cyclophosphamide for 6 cycles	Vincristine, doxorubicin, and cyclophosphamide for additional 8 cycles; no treatment	2
Midlands Small Cell Lung Cancer Group (1986) [24]	32	Limited disease	Vincristine, doxorubicin, and cyclophosphamide for 6 cycles	Vincristine, doxorubicin, and cyclophosphamide for additional 8 cycles; no treatment	2
Han JY (2008) [25]	45	Extensive disease	Irinotecan plus cisplatin for 8 cycles	Irinotecan plus cisplatin for additional 6 cycles; no treatment	3
NH Hanna (2002) [26]	144	Extensive disease	Etoposide, ifosfamide and cisplatin for 4 cycles	Etoposide for additional 3 cycles; no treatment	1
JM Beith (1996) [27]	129	Limited disease and extensive disease	Cisplatin and etoposide (EP) for 4 cycles, plus cranial and local radiotherapy.	Vincristine, doxorubicin and cyclophosphamide for 10 cycles; no treatment	2
The “Petites Cellules” Group (1992) [28]	79	Limited disease and extensive disease	lomustine, cyclophosphamide, doxorubicin and etoposide for 6 cycles	cyclophosphamide, doxorubicin and etoposide for additional 6 cycles; no treatment	1

### Effects of maintenance chemotherapy

**Figure 2:**
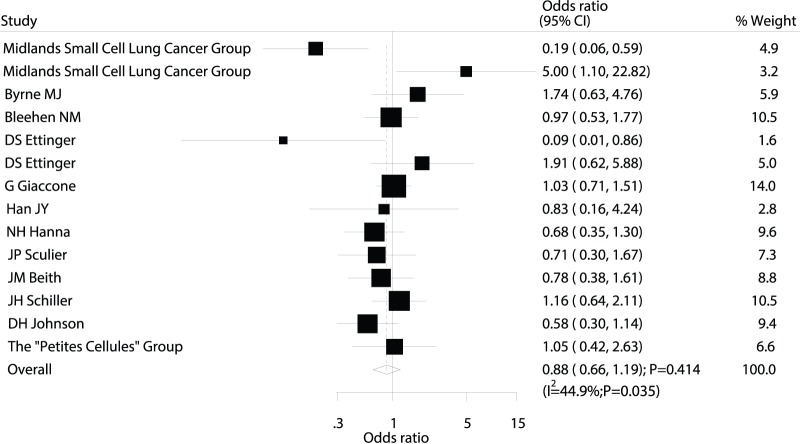
Comparison of 1-year mortality (death from any cause) between maintenance chemotherapy and observation.

Data on the effect of maintenance chemotherapy on 1-year mortality were available from 14 trials [6]–[8], [10]–[13], [24]–[28], which included 1806 patients and reported 1037 death events. Overall, the pooled OR showed a 12% reduction in mortality, but this decrease was not statistically significant (OR: 0.88; 95% CI: 0.66–1.19; P = 0.414; Figure 2). Heterogeneity was observed in the magnitude of the effect across the trials (P = 0.035). However, after sequentially excluding each trial from all pooled analyses, we found that the overall results were not affected by the exclusion of any individual trial.

**Figure 3:**
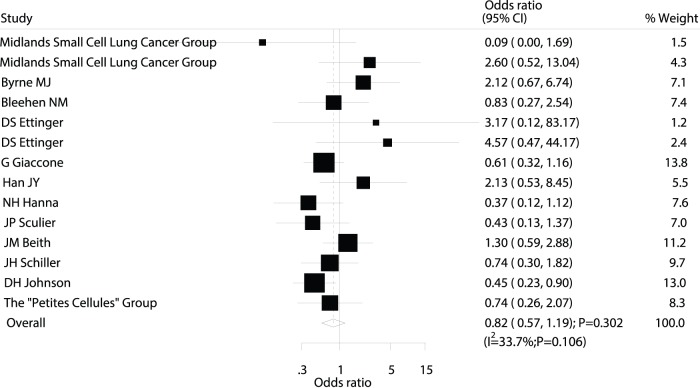
Comparison of 2-year mortality (death from any cause) between maintenance chemotherapy and observation.

Data on the effect of maintenance chemotherapy on 2-year mortality were available from 14 trials [6]–[8], [10]–[13], [24]–[28], which included 1806 patients and reported 1543 death events. No differences in 2-year mortality were observed between patients receiving maintenance chemotherapy and those undergoing observation (OR; 0.82; 95%CI: 0.57–1.19; P = 0.302; Figure 3). Heterogeneity was again observed in the magnitude of the effect across the included trials. As before, sequential exclusion of each trial from all pooled analyses showed that the results were not affected by exclusion of any individual trial.

**Figure 4:**
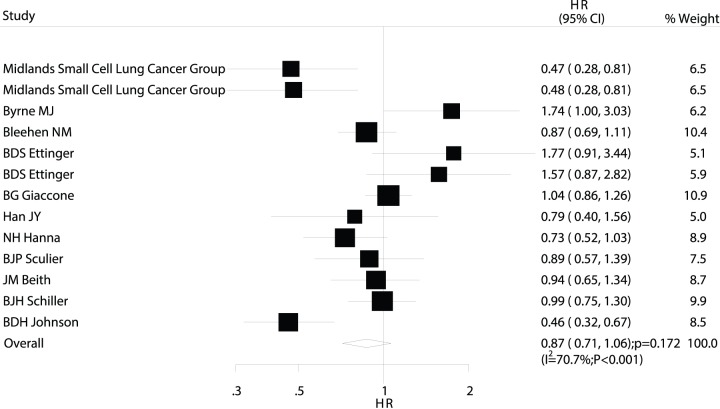
Comparison of overall survival (OS) between maintenance chemotherapy and observation.

Data on OS were available from 13 trials [6]–[8], [10]–[13], [24]–[27]. The trial by the Petites Cellules Group [28] was excluded from our analysis of OS in our study because the author could not provide OS survival data. Overall, maintenance chemotherapy was associated with a 13% improvement in OS, but the difference was not statistically significant (HR: 0.87; 95% CI: 0.71–1.06; P = 0.172; Figure 4). Although substantial heterogeneity may exist in the HRs for OS across the individual trials, we noted that the results were not affected by exclusion of any specific trial.

**Figure 5:**
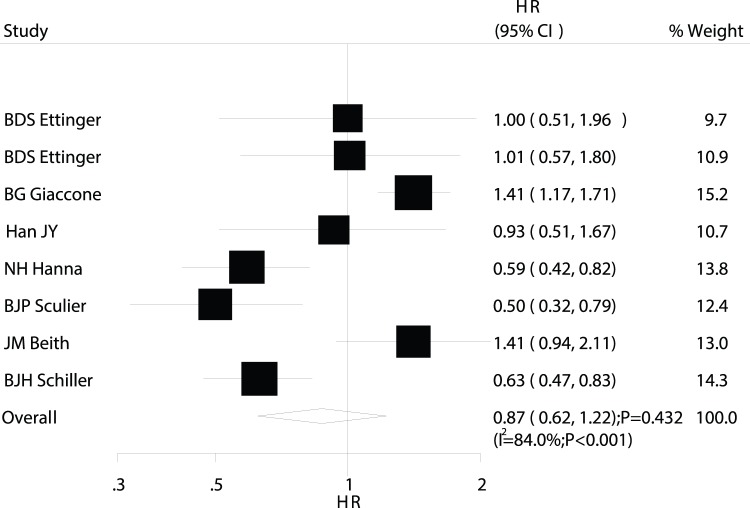
Comparison of progression-free survival (PFS) between maintenance chemotherapy and observation.

Data on PFS were available from 8 trials [8], [10]–[12], [25]–[27]. Overall, maintenance chemotherapy was associated with a 13% improvement in PFS, but the difference was not statistically significant (HR, 0.87; 95%CI: 0.62–1.22; P = 0.432; Figure 5). We also noted substantial heterogeneity in the magnitude of the effect across the included trials. As in the analyses of mortality and OS, sequential exclusion of each trial did not affect the overall results.

### Subgroup analyses

Subgroup analyses were conducted for 1-year mortality, 2-year mortality, OS, and PFS to minimize the consequences of heterogeneity among the included trials. We noted that maintenance chemotherapy was associated with a reduction in 2-year mortality if there were over 100 patients in the study or if patients were managed using the switch strategy. Furthermore, maintenance chemotherapy was associated with a clinically and statistically significant improvement in PFS for trials published after 2000, as well as for patients with extensive disease. Finally, patients managed using the continuous strategy appeared to be at a disadvantage with regard to PFS when compared with patients undergoing observation. No other significant differences were identified between the efficacy of maintenance chemotherapy and observation, when based on additional subset factors (Table 2).

**Table 2:** Subgroup analysis for 1-year mortality, 2-year mortality, OS, and PFS.

Outcomes	Group	OR or HR with their 95%CI	P value	heterogeneity (%)			P value for heterogeneity
**1-year mortality**	**Publication year**						
	Before 2000	0.88 (0.60–1.29)	0.506	54.9			0.014
	After 2000	0.90 (0.59–1.39)	0.646	0			0.485
	**Number of patients**						
	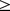 100	0.90 (0.72–1.13)	0.366	0			0.582
	<100	0.89 (0.44–1.83)	0.758	64.7			0.006
	**Stage of disease**						
	Limited disease	1.48 (0.46–4.81)	0.514	74.9			0.019
	Extensive disease	0.66 (0.32–1.33)	0.241	63.8			0.017
	**Chemotherapy regimes**						
	Continuous strategy	0.98 (0.59–1.61)	0.927	58.7			0.013
	Switch strategy	0.78 (0.58–1.06)	0.115	0			0.612
**2-year mortality**	**Publication year**						
	Before 2000	0.85 (0.55–1.31)	0.462		36.8		0.104
	After 2000	0.77 (0.32–1.85)	0.557		46.4		0.155
	**Number of patients**						
	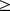 100	0.66 (0.46–0.93)	0.016		5.8		0.379
	<100	1.22 (0.61–2.43)	0.581		36.7		0.136
	**Stage of disease**						
	Limited disease	1.19 (0.34–4.11)	0.783		73.2		0.024
	Extensive disease	0.89 (0.37–2.14)	0.786		43.9		0.113
	**Chemotherapy regimes**						
	Continuous strategy	1.11 (0.64–1.90)	0.715		31.3		0.168
	Switch strategy	0.62 (0.39–0.99)	0.045		27.8		0.236
**OS**	**Publication year**						
	Before 2000	0.88 (0.68–1.14)	0.348			77	<0.001
	After 2000	0.87 (0.71–1.07)	0.181			0	0.380
	**Number of patients**						
	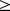 100	0.83 (0.67–1.03)	0.087			70	0.005
	<100	0.95 (0.62–1.45)	0.815			75	0.001
	**Stage of disease**						
	Limited disease	0.72 (0.32–1.60)	0.414			88	<0.001
	Extensive disease	0.92 (0.66–1.29)	0.648			67	0.010
	**Chemotherapy regimes**						
	Continuous strategy	0.95 (0.72–1.27)	0.741			72	0.001
	Switch strategy	0.78 (0.59–1.02)	0.074			67	0.016
**PFS**	**Publication year**						
	Before 2000	1.02 (0.69–1.52)	0.909			78	0.001
	After 2000	0.64 (0.53–0.79)	<0.001			0	0.415
	**Number of patients**						
	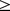 100	0.93 (0.57–1.52)	0.765			91	<0.001
	<100	0.79 (0.54–1.15)	0.226			44	0.150
	**Stage of disease**						
	Limited disease	-	-			-	-
	Extensive disease	0.72 (0.58–0.89)	0.003			21	0.282
	**Chemotherapy regimes**						
	Continuous strategy	1.27 (1.04–1.54)	0.018			8	0.354
	Switch strategy	0.71 (0.47–1.07)	0.103			80	0.002

### Publication bias

**Figure 6:**
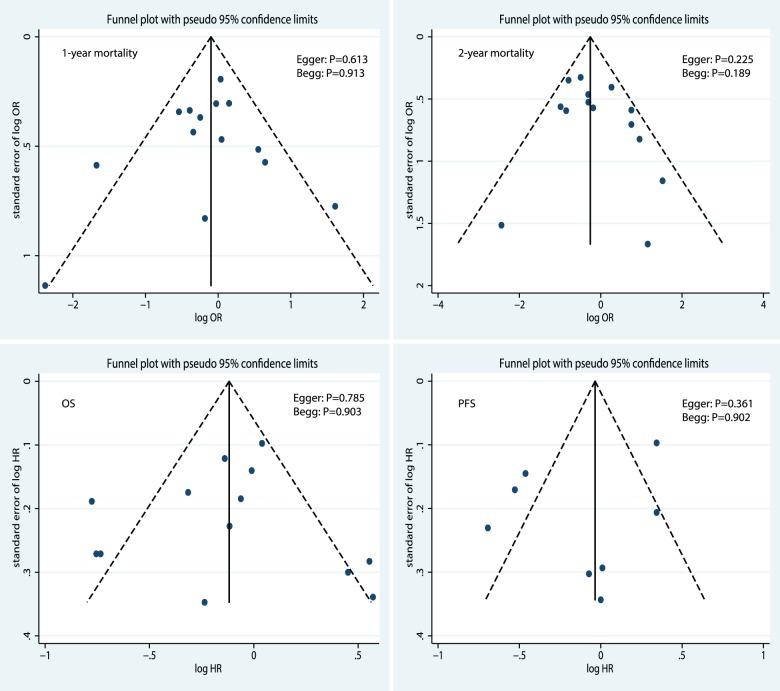
Funnel plots for 1-year mortality, 2-year mortality, OS, and PFS.

The Egger test, Begg test, and funnel plot (Figure 6) showed no evidence of publication bias for 1-year mortality (Egger test, P = 0.613; Begg test, P = 0.913), 2-year mortality (Egger test, P = 0.225; Begg test, P = 0.189), OS (Egger test, P = 0.785; Begg test, P = 0.903), or PFS (Egger test, P = 0.361; Begg test, P = 0.902).

## Discussion

A growing number of trials have evaluated the role of maintenance chemotherapy in the treatment of patients with SCLC, for which clinicians have few evidence-based treatment regimens. Trials of maintenance chemotherapy have arrived at various, divergent conclusions, suggesting that a meta-analysis could clarify the role of maintenance chemotherapy for patients with SCLC. In this updated comprehensive quantitative review, we included over 1806 patients with SCLC, incorporating a broad range of baseline characteristics. The present study suggests that maintenance chemotherapy does not have an effect on 1-year mortality, 2-year mortality, OS, or PFS when compared with observation. Additionally, subgroup analyses indicated that patients who received maintenance chemotherapy were at significantly reduced the risk of 2-year mortality in trials with over 100 patients and in trials using the switch strategy. Maintenance chemotherapy was found to significantly improve PFS in trials published after 2000 as well as in patients with extensive disease. However, patients who were managed using the continuous strategy appeared to be at a disadvantage with regard to PFS when compared with patients who underwent observation.

The Midlands Small Cell Lung Cancer Group (MSCLCG) trial [24] found that patients who received maintenance chemotherapy lived significantly longer than those who underwent observation. However, a trial by Byrne et al. [6] suggested that the OS of patients randomized to receive maintenance therapy was inferior to the OS of those randomized to undergo observation. In the present study, we found no significant differences between maintenance chemotherapy and observation in terms of OS or 1-year mortality. However, the nonsignificant results of our subgroup analysis were consistent with these previous studies because the MSCLCG trial [24] was restricted to patients with extensive SCLC and the Byrne et al. trial [6] was restricted to patients with limited SCLC. Therefore, we suggest maintenance chemotherapy may have a beneficial effect on OS in some subpopulations, but the extent of benefit may be diluted or negated on inclusion of other subpopulations.

A trial by Hanna et al. [26] suggested that patients with extensive SCLC who received an additional 3 months of oral etoposide had significantly improved PFS. The European Lung Cancer Working Party Study [11] found that PFS was significantly improved by maintenance chemotherapy, but OS was not significantly increased. A trial by Johnson et al. [13] indicated that the switch strategy was superior to the continuous strategy in terms of the 2-year mortality of patients with SCLC. These results are in agreement with the findings of our meta-analysis. Our study demonstrated that benefits could be achieved by using the switch strategy in patients with extensive SCLC. We additionally noted that patients who were managed using the continuous maintenance chemotherapy strategy derived less benefit than patients who underwent observation. It is possible that maintenance chemotherapy has greater efficacy for extensive SCLC than for limited SCLC, which could explain the lack of benefit from continuous maintenance in the overall study population. Moreover, we found that patients who were managed using the switch strategy appeared to derive greater benefit than those who were managed using the continuous strategy.

Substantial heterogeneity existed in the magnitude of the effect across the included trials. One explanation of this heterogeneity could be that patients in different trials had different stages of disease and received different chemotherapy regimens. We conducted subgroup analysis to minimize the consequences of heterogeneity across the included studies and, additionally, to evaluate the efficacy of maintenance therapy in specific subpopulations. A variety of studies has already demonstrated that stage of disease and chemotherapy regimens are prognostic factors. Additionally, different publication years implied different standards of medical care and quality of treatment, which could modify the treatment effect. Finally, small sample sizes contribute to broader confidence intervals and, perhaps consequently, no studies with small sample sizes showed significant differences in treatment outcomes. Therefore, year of publication, number of patients, stage of disease, and maintenance chemotherapy regimen were studied as important factors that could alter the interpretation of our results. Our subgroup analyses suggested that maintenance chemotherapy was associated with a reduced risk of 2-year mortality in studies with over 100 patients. We also found that maintenance chemotherapy was associated with clinically and statistically significant improvements in PFS for trials published after 2000. These findings could be explained as follows: (1) A small sample size limited us to investigating intrinsic effects and lead to broad confidence intervals. (2) Medical care and treatment levels, which naturally change over the years, may play an important role in improvements in PFS.

Multiple testing is an inherent limitation to any meta-analysis that is based on published evidence and evaluates the roles of interventions in specific populations. The limitations of multiple testing arise because each individual study includes different baseline characteristics and subpopulations. In the present study, subgroup analyses were only conducted for between-study hypothesis, rather than within-study hypothesis, because original data and data on individual patients were unavailable. However, these subgroup analyses have acceptable validity because we only stratified data according to 4 factors. We conducted subgroup analyses to provide a relative assessment that compared maintenance chemotherapy with observation and that evaluated the efficacy of maintenance therapy in specific subpopulations.

Most of our study's findings were consistent with a meta-analysis of SCLC treatments that was published in 2010 [14], which suggested that maintenance and consolidation therapies failed to improve the survival outcomes of patients with SLCL. However, that study [14] incorporated not only chemotherapy but also treatment with interferons and other biological agents to evaluate maintenance or consolidation therapy in patients with SCLC. Although that meta-analysis detected a statistically significant reduction in mortality in trials that assessed the efficacy of chemotherapy and interferon-α therapy, it failed to evaluate the efficacy of maintenance therapy in subpopulations. In the present study, we have updated the results of this prior meta-analysis and additionally conducted subgroup analyses to investigate the factors that could substantially modify the interpretation of our data. The results of our meta-analysis are promising because they favor the use of maintenance chemotherapy in some subpopulations, such as patients with extensive disease, and suggest that some maintenance chemotherapy regimens are more effective than others. Indeed, we found that the switch chemotherapy strategy was superior to the continuous chemotherapy strategy.

There are several technical limitations to this meta-analysis. First, we made inherent assumptions by using pooled data in our meta-analyses. (These data were either previously published or provided by study authors.) Individual patient data and original data were unavailable, which prevented us from performing more detailed analyses and obtaining more comprehensive results. Second, the substantial heterogeneity that was observed across included trials could not be eliminated by subgroup analysis. Third, maintenance chemotherapy administered in the trials included different regimens, which prevented us from evaluating the association between the type of maintenance chemotherapy and survival outcomes. Fourth, data on adverse events were rarely available; therefore, no conclusions could be drawn regarding adverse events. Fifth, multiple testing presents another limitation to our study. Finally, the Jadad score emphasizes the importance of blinding, but neither patients nor doctor can be blinded to the choice of chemotherapy or observation. Further, studies included in our meta-analysis did not provide information about whether the data manager was blinded. Therefore, we regarded studies with a score of 3 or greater as high-quality studies.

In conclusion, we found that maintenance chemotherapy has no significant effects on 1-year mortality, 2-year mortality, OS, or PFS. Subgroup analyses suggested that maintenance chemotherapy could play an important role in 2-year mortality for trials involving more than 100 patients or for patients who are managed using the switch strategy. Additionally, patients who are managed using the switch strategy appeared to derive superior benefits, as compared with those who were managed using the continuous chemotherapy strategy. In future trials, promising maintenance chemotherapy regimens should be investigated for patients with extensive SCLC. Future trials should also consider the duration of maintenance chemotherapy. Through such trials, the optimal duration of treatment and optimal treatment regimens might both be confirmed. We suggest that ongoing trials be improved as follows: adverse events should be recorded and reported formally and additional treatment regimens and the duration of treatment should be taken into consideration when evaluating survival outcomes.

## Supporting Information

Table S1 **PRISMA Checklist.**
(DOC)
